# Colored Medical Students at Paris

**Published:** 1852-11

**Authors:** 


*Colored Medical Students at Paris.*—Professor Eve, of Nashville, Tenn., relates the following anecdote in a letter from Paris:

"A few days before leaving Paris, we had quite a *nice* little professional anecdote to occur in one of its hospitals. France is essentially democratic, however she may tolerate a despotic ruler. All classes of society, and all *colors*, too, mingle freely there. Among the students of Velpeau, is one as black as can be, who observing a South Carolinian recently arrived, took peculiar and persevering pleasure in exhibiting the interests of the great charité hospital, much to the annoyance of our young countryman. During an operation, the negro asked him where he was from. Charleston, South Carolina, was the reply; when the black promptly observed, 'Oh! ah! then we are *fellow-students* and *fellow-patriots;* for I am from Boston, Massachusetts.'"—*Nashville Jour. of Med. and Surg.*

